# Quantifying capture stress in free ranging European roe deer (*Capreolus capreolus*)

**DOI:** 10.1186/s12917-017-1045-0

**Published:** 2017-05-10

**Authors:** Nikolaus Huber, Sebastian G. Vetter, Alina L. Evans, Petter Kjellander, Susanne Küker, Ulrika A. Bergvall, Jon M. Arnemo

**Affiliations:** 1 Department of Integrative Biology and Evolution, Research Institute of Wildlife Ecology, University of Veterinary Medicine, Vienna, Austria; 2 Department of Forestry and Wildlife Management, Faculty of Applied Ecology and Agricultural Sciences, Inland Norway University of Applied Sciences, Campus Evenstad, NO-2418 Elverum, Norway; 3 Department of Ecology, Swedish University of Agricultural Sciences, Grimsö Wildlife Research Station, SE-730 91 Riddarhyttan, Sweden; 4 Department of Clinical Research and Veterinary Public Health, Veterinary Public Health Institute, Vetsuisse Faculty, University of Bern, Bern, Switzerland; 5 Department of Zoology, Stockholm University, SE-106 91 Stockholm, Sweden; 6 Department of Wildlife, Fish and Environmental Studies, Faculty of Forest Sciences, Swedish University of Agricultural Sciences, SE-901 83 Umeå, Sweden

**Keywords:** Wildlife, Stress, Leukocyte coping capacity, Coping style, Cortisol

## Abstract

**Background:**

To understand and reduce the concomitant effects of trapping and handling procedures in wildlife species, it is essential to measure their physiological impact. Here, we examined individual variation in stress levels in non-anesthetized European roe deer (*Capreolus capreolus*), which were captured in box traps and physically restrained for tagging, biometrics and bio-sampling. In winter 2013, we collected venous blood samples from 28 individuals during 28 capture events and evaluated standard measurements for stress (heart rate, body temperature, neutrophil to lymphocyte ratio, lactate and total cortisol). Additionally, we assessed stress using the immunological tool, Leukocyte Coping Capacity (LCC), a real-time proxy for stress measuring oxygen radical production by leukocytes. Finally, the behavioral response to handling was recorded using a scoring system.

**Results:**

LCC and therefore stress levels were negatively influenced by the time animals spent in the box trap with human presence at the capture site prior to handling. In contrast, none of the classical stress measures, including total cortisol, nor the behavioral assessment, were correlated with the stressor tested (time of human presence prior to handling) and thus did not provide a clear depiction regarding the extent of the animals short-term stress response.

**Conclusions:**

Overall our study verifies the LCC as a strong method to quantify short-term stress reactions in wildlife. Moreover, our results clearly show that human presence at the trapping site prior to handling should be kept to an absolute minimum in order to reduce stress levels.

**Electronic supplementary material:**

The online version of this article (doi:10.1186/s12917-017-1045-0) contains supplementary material, which is available to authorized users.

## Background

With the growing complexity of anthropogenic environments, the general demands on wildlife management and conservation are constantly increasing [1, 2]. These fields often include captures and it is important to evaluate the animal welfare implications and to verify that the methods used have not affected the quality of the resulting scientific data [3, 4]. Animal welfare is mostly defined as the well-being of animals and is closely linked to the capability of the individual to cope with sudden situations or changes in its environment [5]. If an animal has difficulties to adapt or is not able to cope with the prevalent situation, it becomes stressed [6]. Stress levels, however, are not only relevant in terms of animal welfare, but also bias various physiological and behavioral measures [7]. In order to increase animal welfare and data quality it is essential to assess and quantify how management and research interventions (i.e. capture and handling) affect stress levels [8]. Stress-responses in vertebrates are primary mediated via two neuro-endocrine regulatory systems, stimulating physiological adaption and behavior. First, the sympathetic nervous system (SNS) produces immediate responses such as the fight or flight response. Activation of the SNS triggers the release of catecholamines within milliseconds after onset of a stressor [9]. Second, the hypothalamic-pituitary-adrenal (HPA) axis responds and controls the secretion of glucocorticoids. This response is slower (within minutes) and acts on many metabolic and physiological regulatory systems to keep essential bio-regulatory mechanisms within a certain range [10, 11].

Despite extensive research, stress remains a problematic concept because stress reactions are multidimensional and context dependent [12]. Additionally, stress responses display a large individual variation [9], are influenced by season as well as time of day [13] and are caused by a great variety of stressors [14]. Consequently, stress reactions are difficult to measure and assess, particularly with small sample sizes as it is often the case in field studies [15].

Currently stress responses are assessed by various techniques [16–18]. Previous work on wildlife species has focused mainly on glucocorticoid concentrations to define stress [19]. It becomes increasingly apparent, however, that this approach could be misleading due to the wide array of factors influencing the release and efficiency of glucocorticoids [20, 21]. Additionally, there is evidence that they are not stress hormones per se, but rather anti-stress hormones promoting recovery from stress reactions [10, 22]. Therefore, the use of glucocorticoids as a single biomarker for measuring stress is questionable, especially when assessing short-term stress reactions [12, 23]. Furthermore, alongside other classical stress parameters including heart rate and body temperature, which are viable indicators for stress [24, 25], alterations in behavior have been used to determine stress levels in free-ranging wildlife [26]. However, also the behavioral approach is under scientific debate, as the source of the underlying physiological responses are problematic [27]. Hence, there is a need for new, practical tools linking and complementing classical stress parameters towards a more comprehensive description and interpretation of stress responses.

There is growing evidence of the effects of stress on parts of the innate immune system [28–30] indicating that it is possible to quantify stress by directly measuring immune responses [31, 32]. White blood cells, more specifically neutrophil granulocytes, of stressed individuals show a substantially decreased capacity to produce reactive oxygen species (ROS) compared to less stressed animals. Applying the technique called Leukocyte Coping Capacity (LCC) facilitates a quantitative assessment of stress responses by measuring neutrophil ROS production in real time [31, 33].

In the present study, we tested the validity of LCC together with several classical key stress parameters (heart rate, body temperature, neutrophil to lymphocyte ratio (N:L), lactate, total cortisol and animal behavior), to quantify the short-term stress of capture in one of the most intensively managed [34] free-ranging wild ungulates in Europe, the European roe deer (*Capreolus capreolus*). Moreover, we investigated whether LCC measures could be linked to these classical and commonly used stress parameters.

We hypothesized that, in contrast to classical stress parameters, the LCC technique facilitates a clear and quantitative assessment of stress in roe deer. Here we tested human presence at the capture site prior to handling as the stress eliciting factor (see Methods). We predicted that (i) LCC will allow to quantify the extent of the stress reaction caused by this stressor [31, 33] and (ii) individuals experiencing high stress levels will exhibit a lower LCC compared to less stressed individuals. Further, we predicted that (iii) due to large individual variation neither the behavior of the animals nor classical stress measures (heart rate, body temperature, N:L ratio, lactate, total cortisol) will reflect the magnitude of short-term stress accurately [20, 21, 27]. However, regarding a possible link between classical stress parameters and LCC we expected that stressed individuals with elevated classical stress parameters would have a low LCC. In short, LCC would be negatively correlated with classical stress proxies.

## Methods

### Definition of stress and stressor

In this study we defined the situation of being trapped in combination with human presence at the capture site as the stressor of interest eliciting a short-term stress response [9]. The activation of both stress axes and the associated physiological changes are referred to as stress.

### Study area and data collection

The study was conducted at the Grimsö Wildlife Research Area (GWRA, 130 km^2^) in southcentral Sweden (59°40′N, 15°25′E). The predominant landscape is commercially managed coniferous forest (for details see [35]).

The roe deer population of this area has been intensively studied since 1973 [35] with >3500 captures carried out for various research studies and monitoring purposes. To assess stress reactions in the context of these capture events, where animals are handled without the use of anesthetics, 28 capture events of 28 individuals (18 females, 10 males) were included in this study. Captures took place in winter 2013, in two sampling periods (January 17^th^ – January 30^th^ and March 4^th^ – March 24^th^). These periods did not overlap with the phase of rutting, parturition [36], or male territoriality [37]. Animals were caught using box traps (L6 Rådjursfälla M/Öster Malma, dimensions: 130 × 62.5 cm and 100 cm high), baited with pelleted forage produced for semi-domesticated reindeer (*Rangifer tarandus tarandus*) (Renfor, Lantmännen, Nyköping, Sweden). Every trapping site was equipped with two traps which were set in the evenings and checked early the next morning. The front of the trap was closed with wooden bars enabling the animals to see their conspecifics next to the trap. The hatch and sidewall of traps were closed with Masonite® plates blocking visual contact with approaching humans. However, the trapped animal could potentially hear or smell an approaching person. Traps were placed at varying distances from the road (<200 m), but all were visible from the road. In rare cases two individuals were caught at the same trapping site leading to a prolonged time span of human presence prior to handling for the second animal. We recorded the timespan between the arrival at the trapping site by car (on the road) until the start of the actual handling procedure to account for the human presence prior to handling and a potential further onset of a stress response.

Animals were removed from the trap and physically restrained in lateral recumbency on the ground during handling and marking. Once restrained, body temperature and heart rate were measured and a blood sample was obtained immediately (see below). Subsequently, morphological measures were taken and the animals were sexed and weighed.

Additionally, individual behavior was assessed during handling and upon release. Handling behavior was evaluated based on resistance to handling and vocalization of the animal, with scores ranging from 0 (calm, displaying no resistance) to 4 (extreme resistance and almost impossible to handle) (Table 1). Behavior upon release was assessed with scores from 0 to 2, relating to flight speed and the number of intermediate stops (Table 1). The behavioral observation was performed continuously by the same experienced person (L. Jäderberg, >2500 captures). To avoid adverse effects due to physical restraint during the handling procedure of the non-anesthetized animals [38, 39], handling time was minimized. All deer were physically examined by a veterinarian and appeared healthy.

**Table 1:** Behaviors of roe deer recorded during handling and release. The score represents the judgement of an experienced handler (same person for all measures) as to how excited each animal was during handling and release, relative to other individuals in the population, displaying representative combinations of behaviors

Score	Behavior during handling	Behavior upon release
0	Calm. No resistance. No kicking or screaming.	Leaving the place slowly. Stops several times.
1	Calm. Screams not more than twice. Almost no kicking.	Runs away, but stops after a short distance.
2	Intermittent screaming and kicking, but apparently calm.	Runs away without stopping until out of sight.
3	High resistance. Screaming and kicking more, but can be handled.	
4	Extreme resistance. Almost impossible to handle. Impossible to take proper measurements.	

### Blood sampling

Blood samples were taken from the jugular vein in <7 min after animals were taken out of the trap. Evacuated tubes (BD Vacutainer®, BD Diagnostics, Preanalytical Systems, Franklin Lakes, NJ, USA) were used with one 6-ml ethylenediaminetetra-acetic acid (EDTA) (potassium), one 4 ml sodium-heparin tube and four 9-ml serum or gel serum separator tubes. From EDTA samples standard hematological parameters (for details see Additional file 1: Table S1) were analyzed within 24 h of collection. Serum for blood chemistry was separated within 2 h of collection by centrifugation at 1500 x G for 10 min and stored in cryogenic vials (Nalgene, Nalgene Company, Rochester, New York, USA) at −20 °C. Total cortisol levels of all individuals were measured from these serum samples. All samples were analyzed within 3 months of storage (Additional file 2: Table S2) at the Clinical Chemistry Laboratory, Faculty of Veterinary Medicine and Animal Science, Swedish University of Agricultural Sciences, Uppsala, Sweden. Lactate levels were measured from EDTA blood samples within 15 min after collection using the portable Lactate Pro® lactate analyzer (KBK, Arkray, Japan).

### LCC measurements

To measure unstimulated blood chemiluminescence levels, providing information on the individual baseline level of ROS, we immediately transferred 10 μl of heparinized whole blood into a silicon antireflective tube (Lumivial, EG & G Berthold, Germany). We added 90 μl of 10^−4^ mol l^−1^ luminol (5-amino-2,3-dihydrophthalazine-1,4-dione; VWR International, Stockholm, Sweden) which was dissolved in dimethyl sulfoxide (DMSO; VWR International, Stockholm, Sweden) and diluted with phosphate-buffered saline (PBS, pH 7.4). 10 μl of PBS were added and the tube was shaken gently for mixing. The lumigenic substrate, Luminol, produces chemiluminescence when combined with an oxidizing agent, producing a low-intensity light reaction [40]. To measure full blood chemiluminescence produced in response to a secondary challenge (the first challenge was the stress reaction in vivo), a second tube was prepared in parallel as described above but 10 μl of 10^−5^ mol l^−1^ phorbol 12-myristate 13-acetate (PMA; VWR International, Stockholm, Sweden) was added instead of 10 μl PBS [30]. The higher the extent of the first challenge (i.e., the stress reaction of the animal), the lower the chemiluminescence response to the artificial secondary challenge is going to be. In other words, low LCC values indicate high stress levels and vice versa.

Blood chemiluminescence for each tube was assessed every 5 min for a total of 30 s over a period of 30 min and expressed in relative light units (RLU), using a portable high sensitivity chemiluminometer (Junior LB 9509, EG & G Berthold, Germany). All measurements were carried out in the field inside a car, ensuring stable conditions above 15 °C and were performed immediately after the blood sample was collected. When not in the chemiluminometer, tubes were incubated at 37 °C in a lightproof water bath. The texture and adhesiveness of the cell microenvironment is essential for the in vivo determination of cell reactivity [41]. Using the LCC technique, working with minimal diluted whole blood without further manipulation of the cell and immediate performance of the analysis, ensures the structural integrity and morphology of the cell.

### Statistical analysis

**Fig. 1:**
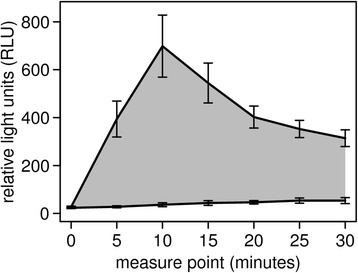
Leukocyte Coping Capacity (LCC) curve (measured every 5 min for 30 s over 30 min) for European roe deer (*n* = 28), captured in box traps. The lower line represents basal levels of reactive oxygen species (ROS) whereas the upper line represents PMA stimulated samples. The grey shaded area indicates the integral of the area under the curve (auc). Data points represent mean LCC levels (in relative light units), with error bars showing the standard error of the mean (S.E.M) for each time point

All statistical analyses were performed in R.3.0.2 [42]. To analyze whether the LCC was reflecting individual stress levels, we tested whether the LCC-peak (see Fig. 1) as well as the integral of the LCC curve (area under the curve, auc; Fig. 1) were affected by the time the animal spent in the trap with human presence at the capture site prior to handling (waiting time), the animal’s handling score and, its release score. Given that these two behavioral scores might represent proxies for an animal’s coping style [43] which in turn could affect its response to a stressor we also included the pairwise interactions of waiting time with the behavioral scores in the two negative binomial models (R-package “MASS” [44]), which were additionally corrected for the total number of neutrophils (both models *n* = 24).

Preliminary analyses showed that including sampling period as a random effect in the models for correction did not affect the results and thus our conclusions. Given the small sample sizes we used the more parsimonious model and removed this parameter from the analysis beforehand. This also holds for all models presented below.

In order to test whether the stress reaction caused by human presence at the trap is also reflected by commonly used stress indicators, we tested whether heart rate, body temperature, total cortisol, N:L ratio or lactate levels were affected by waiting time, the animal’s handling score, its release score, or the pairwise interactions of waiting time with these two behavioral scores. For this purpose we performed two negative binomial models, on cortisol (*n* = 26) and N:L ratio (*n* = 24), and three linear models (R-package “stats” [45]), on heart rate (*n* = 27), body temperature (*n* = 27), and lactate levels (*n* = 19).

For all models, we determined relevant explanatory variables by comparing all nested models in a model selection table based on Akaike’s Information Criterion corrected for small sample size (AICc; [46, 47]) from which we selected the model with the lowest AICc. Model selection tables, including all models with a ΔAICc <4 and the Null-model only containing the intercept, are shown in the Additional file 3: Tables S3-S9. Full models of all linear models showed no evidence for serious deviations from normality according to the inspection of the distribution of residuals by means of histograms and quantile-quantile plots.

Finally, we tested pairwise correlations between all investigated stress indicators (all explanatory variables described above plus the two behavioral indices) using Pearson correlation tests. In order to correct for multiple testing we corrected the *p*-values using the Benjamini-Hochberg method [48]. However, as most *p*-values leveled off at around 1.0 after correction and were therefore not informative, we additionally report the uncorrected *p*-values in the results (Table 2).

**Table 2:** Pairwise Pearson correlations between potential stress indicators (i.e., LCC-peak, LCC-auc, N:L ratio, cortisol, lactate, heart rate, body temperature, handling score and release score)

	LCC-auc	N:L ratio	Cortisol	Lactate	Heart rate	Body temp.	Handling score	Release score
LCC-peak	***r*** **= 0.95** ***P*** **< 0.001** ***p*** **< 0.001** ***N*** **= 28**	*r* = 0.33 *P* = 0.87 *p* = 0.11 *N* = 25	*r* = −0.08 *P* = 1.00 *p* = 0.70 *N* = 27	*r* = −0.01 *P* = 1.00 *p* = 0.95 *N* = 20	*r* = 0.14 *P* = 1.00 *p* = 0.51 *N* = 28	*r* = 0.10 *P* = 1.00 *p* = 0.60 *N* = 28	*r* = 0.31 *P* = 1.00 *p* = 0.88 *N* = 27	*r* = −0.16 *P* = 1.00 *p* = 0.44 *N* = 27
LCC-auc		*r* = 0.42 *P* = 0.30 *p* = 0.04 *N* = 25	*r* = −0.07 *P* = 1.00 *p* = 0.75 *N* = 27	*r* = 0.07 *P* = 1.00 *p* = 0.75 *N* = 20	*r* = 0.14 *P* = 1.00 *p* = 0.47 *N* = 28	*r* = 0.13 *P* = 1.00 *p* = 0.51 *N* = 28	*r* = 0.08 *P* = 1.00 *p* = 0.71 *N* = 27	*r* = −0.14 *P* = 1.00 *p* = 0.48 *N* = 27
N:L ratio			*r* = −0.20 *P* = 1.00 *p* = 0.34 *N* = 24	*r* = 0.20 *P* = 1.00 *p* = 0.44 *N* = 17	*r* = −0.17 *P* = 1.00 *p* = 0.43 *N* = 25	*r* = 0.20 *P* = 1.00 *p* = 0.34 *N* = 25	*r* = 0.15 *P* = 1.00 *p* = 0.48 *N* = 24	*r* = −0.12 *P* = 1.00 *p* = 0.58 *N* = 24
Cortisol				*r* = 0.22 *P* = 1.00 *p* = 0.34 *N* = 20	*r* = −0.26 *P* = 1.00 *p* = 0.19 *N* = 27	*r* = 0.41 *P* = 0.26 *p* = 0.03 *N* = 27	*r* = 0.04 *P* = 1.00 *p* = 0.86 *N* = 26	*r* = 0.32 *P* = 0.94 *p* = 0.12 *N* = 26
Lactate					*r* = −0.21 *P* = 1.00 *p* = 0.38 *N* = 20	*r* < 0.01 *P* = 1.00 *p* = 0.98 *N* = 20	*r* = 0.39 *P* = 0.82 *p* = 0.10 *N* = 19	*r* = 0.08 *P* = 1.00 *p* = 0.75 *N* = 19
Heart rate						*r* = −0.19 *P* = 1.00 *p* = 0.32 *N* = 28	*r* = 0.37 *P* = 0.46 *p* = 0.06 *N* = 27	*r* = −0.21 *P* = 1.00 *p* = 0.28 *N* = 27
Body temp.							*r* = −0.19 *P* = 1.00 *p* = 0.33 *N* = 27	*r* = 0.14 *P* = 1.00 *p* = 0.48 *N* = 27
Handling score								*r* = −0.21 *P* = 1.00 *p* = 0.30 *N* = 27

The correlation coefficient (*r*), the corrected (*P*) and uncorrected *p*-values (*p*), and the sample size (*N*) are shown

Significant correlations are highlighted bold

## Results

No mortalities or injuries occurred during captures or handling. Total handling time ranged from 7 to 29 min with a mean handling time of 14 (± standard deviation (SD)) 6.8 min. Mean values for complete blood counts and biochemistry parameters were within the reference ranges for European roe deer [49] (for details see Additional file 1: Tables S1 and Additional file 2: Table S2).

The animals had a mean heart rate (± SD) of 107 (±26) beats per minute and a mean body temperature (± SD) of 38.8 (±0.7) °C. The LCC-peaks occurred at 10 min in 78.5% of the roe deer, with exceptions at 5 (11%), 15 (7%), and 20 min (3.5%) (Fig. 1).

**Fig. 2:**
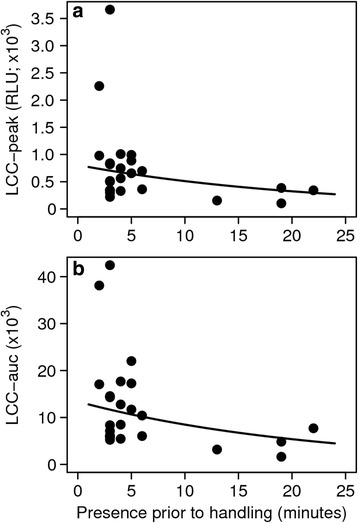
Leukocyte Coping Capacity (LCC) -peak levels (**a**; *n* = 24) and LCC-auc (**b**; *n* = 24) expressed in relative light units (RLU) for European roe deer, captured in box traps, as function of the time the handling team was already present at the capture site prior to the handling procedure. With the increasing time of human presence prior to handling, the LCC response (peak as well as area under the curve, auc) is decreasing, indicating a significant increase of stress in the animals

Besides the number of neutrophils, which was included for correction, the best models for the LCC-peak and the LCC-auc contained only the negative effect of waiting time (peak: estimate ± se = −0.046 ± 0.017, ΔAICc = 2.35, *n* = 24, Fig. 2a, Additional file 3: Table S3; auc: estimate ± se = −0.046 ± 0.014, ΔAICc = 2.69, *n* = 24, Fig. 2b, Additional file 3: Table S4).

The best models for cortisol included waiting time, release score and the interaction of the two (estimate ± se = 0.052 ± 0.021, *n* = 26, Additional file 3: Table S5). The best model on heart rate and lactate levels contained the handling score (estimate ± se = 8.719 ± 4.382, *n* = 27, Additional file 3: Table S6; slope ± se = 1.386 ± 0.803, *n* = 19, Additional file 3: Table S9). However, for those three parameters (cortisol, heart rate and lactate) the Null-model only containing the intercept was very close to those best models (cortisol: ΔAICc = 3.82, heart rate: ΔAICc = 1.42, lactate: ΔAICc = 0.22).

The best models on the N:L ratio as well as on body temperature were the Null-models only containing the intercept (N:L ratio: Additional file 3: Table S7; *n* = 24; body temperature: *n* = 27, Additional file 3: Table S8). The second best models contained the waiting time (estimate ± se = −0.047 ± 0.042) and the handling score (slope ± se = −0.124 ± 0.126), respectively.

In the pairwise comparisons between the different stress parameters, only LCC-peak and LCC-auc were significantly correlated (Table 2).

## Discussion

### The use of LCC to quantify stress in roe deer

Our results support our predictions that the LCC-technique is a suitable technique to quantify short-term stress levels as a result of capture and a short period of subsequent handling in non-anesthetized roe deer (prediction i). In contrast the traditional indicators of stress were not as conclusive (prediction ii). This study presents the first results of LCC for a ruminant species (Fig. 1). Although the exact capture time of the roe deer in this study was unknown and individuals consequently might have spent different timespans within the trap, causing some potential noise, our results clearly demonstrate that LCC reflects the duration of human presences at the capture site.

The obtained LCC responses match indications from previous studies on Atlantic salmon (*Salmo salar*) [50], European badger (*Meles meles*) [31], Rhesus macaque (*Macaca Mulatta*) [51], European water vole (*Arvicola terrestris*) [7], water voles (*Arvicola terrestris*) [52], as well as on humans [33, 53, 54]. Interestingly, estimates of roe deer neutrophil production of reactive oxygen species (ROS) due to secondary challenge peak mostly after five (11%) or 10 min (78.5% of all animals). This finding indicates a considerably faster reactivity of roe deer neutrophils to external stimuli, such as bacterial peptides [55], as well as to stress induced changes in the blood stream [56]. Other species investigated so far reached LCC peak performance only at 15 min (e.g. European badger [31], Brown bear (*Ursus arctos*) [32]). This finding could potentially be explained by the fact that roe deer are prey animals [57, 58] and need to adjust their physiology rapidly in response to a threat by a predator. However, to elucidate the underlying mechanisms, further research is necessary.

Koolhaas et al. [12] highlighted that the term stress should be restricted to conditions which are uncontrollable and unpredictable and as a consequence are potentially life threatening. For prey animals like roe deer, being trapped and unable to escape the presence of a human being, a potential predator, undoubtedly reflects a situation meeting the described conditions and is expected to result in acute psychological stress [59]. Although we do not know the total time the animals spent in the box trap, which could constitute a potential bias in the animals stress response and LCC [60, 61], this stress is conclusively reflected in our LCC measurements. We found that LCC in roe deer significantly decreases with increasing time of humans being present at the capture site (i.e., waiting time; Fig. 2a, b). Correspondingly this confirms the fast change of this immune parameter in consequence of short-term stress. This is supported by findings of Ellard et al. [62] showing that already short-term mental stress causes a significant increase of activated leukocytes in humans, altering their oxidative capacity. Directly related to these findings, Shelton-Rayner et al. [33, 63] showed that acute psychological stress is decreasing LCC values in humans. Thus, our results endorse our first prediction that a stress reaction is already triggered by the presence of humans and not merely by the actual handling procedure.

Two individuals showed extremes in LCC peak values and the LCC-auc response respectively (Fig. 2a, b). Neutrophil ROS production is highly upregulated (>200%) by neutrophil “priming” agents such as chemoattractants (bacterial peptides/proteins), inflammatory cytokines (e.g. tumor necrosis factor alpha) or Toll-like receptor agonists (e.g. endotoxins) [64]. Bacterial infections and/or inflammatory processes increase chemoattractant levels in the blood stream and therefore increase neutrophil ROS production. This could potentially explain the high magnitude in LCC in these two individuals. The extreme nature of the priming effects facilitates a clear distinction to apparently healthy individuals (Fig. 2a, b). Removing these two outliers from the statistical analysis did not change our results.

### Classical stress parameters

The more commonly and more frequently used measures of stress including heart rate [65, 66], body temperature [67], cortisol [19] and leukocyte profiles in terms of the N:L ratio [61, 68] as well as the two behavioral scores neither correlated with LCC values nor with each other and did not reflect our defined stressor. We attribute this lack of correlation to a large individual variation in how animals cope with a stressful situation behaviorally and physiologically [15, 69, 70], impeding the detection of quantitative effects of a stressor, at least at low sample sizes like they are commonly faced in field studies. Moreover, our results are supported by the findings of Esteruelas et al. [32] showing that LCC levels did not correlate with heart rate, N:L ratio nor cortisol concentrations in Scandinavian brown bears. It should be noted, however, that those animals were anesthetized, resulting in additional physiological alterations. As in our findings, the classical parameters measured by Esteruelas et al. [32] indicated that animals were certainly stressed, but likewise did not allow for quantifying stress responses [61]. This, however, is apparently not the case for the LCC. In the case of heart rate and body temperature it may be that these two parameters reflect the very immediate response of the organism to a stressor and that they are therefore not as conclusive towards events occurring several minutes prior to measurement.

Alternatively, this discrepancy between LCC and classical stress parameters could be explained by the characteristic of neutrophils to detect several biochemical alterations in the blood stream linked to stress. Neutrophils provide over 150 different receptors all of which are sensitive to signals of stress in the organism: endocrine factors in the plasma, changes in blood biochemistry and red cell hemodynamics, cytokines as well as changes of products released by the hypothalamic – pituitary – adrenal axis as well as the sympathetic nervous system [71]. Therefore the LCC response may be the cumulative result of the nearly simultaneous shift of all these factors, not allowing a clear correlation with one of the classical stress parameters, especially with low sample sizes.

## Conclusions

Based on our findings, we strongly recommend minimizing both the time the handling teams spend next to restrained (trapped) animals and the handling time to an absolute minimum. This will improve animal welfare and minimize negative effects of stress induced physiological changes on the data collected. The LCC technique proved to be an excellent tool to quantitatively assess short term stressors, even with low sample sizes. The method allows for measuring the stress response in the context of trapped animals and human presence in a quantitative manner. In contrast, this was not possible with the classical stress parameters used in this study.

Due to the rapid changes in LCC in response to short-term stressors, we suggest to additionally analyze catecholamine levels, which could provide an important link between classical stress parameters and the immunological tool of LCC.

## Additional files

Additional file 1: Table S1. Hematology for free-ranging, non-anesthetized European roe deer (*Capreolus capreolus*). (DOCX 34 kb)

Additional file 2: Table S2. Serum chemistry for free-ranging, non-anesthetized European roe deer (*Capreolus capreolus*). (DOCX 40 kb)

Additional file 3: Tables S3-S9. Model selection tables. (DOCX 63 kb)

## Abbreviations

AICc: Akaike’s Information Criterion corrected for small sample size
auc: area under the curve
DMSO: Dimethyl sulfoxide
EDTA: Ethylenediaminetetraacetic acid
GWRA: Grimsö Wildlife Research Area
HPA: Hypothalamic-pituitary-adrenal axis
LCC: Leukocyte Coping Capacity
N:L ratio: Neutrophil-to-lymphocyte ratio
PBS: Phosphate-buffered saline
PMA: Phorbol 12-myristate 13-acetate
RLU: Relative Light Units
ROS: Reactive oxygen species
SD: Standard deviation
S.E.M.: Standard error of the mean
SNS: Sympathetic nervous system
