# Supplementary material for: Quantifying capture stress in free ranging European roe deer (Capreolus capreolus)
Source: BMC Vet Res. 2017 May 10;13:127. doi: 10.1186/s12917-017-1045-0 (PMC5424289; doi:10.1186/s12917-017-1045-0)
Supplement: Supplementary file 1 — Hematology for free-ranging, non-anesthetized European roe deer (Capreolus capreolus). (DOCX 34 kb) [file 12917_2017_1045_MOESM1_ESM.docx]

**Table S1**Hematology for free-ranging, non-anesthetized European roe deer (*Capreolus capreolus*), captured at Grimsö Wildlife Research Area, included in the study. Ranges as well as the Mean ± SD are presented with all animals included.

| Parameter | Range | Mean ± SD | *n* |
| --- | --- | --- | --- |
| Platelet (10^3^/µL) | 168.00 - 602.00 | 344.96 ± 111.24 | 25 |
| RBC (10^12^/L) | 9.60 - 13.80 | 11.55 ± 0.92 | 27 |
| HGB (g/L) | 150.00 - 204.00 | 185.00 ± 11.25 | 27 |
| HCT (L/L) | 0.43 - 0.59 | 0.52 ± 0.03 | 27 |
| MCV (fL) | 41.00 - 50.00 | 45.55 ± 2.48 | 27 |
| MCHC (g/L) | 332.00 - 374.00 | 352.6 ± 8.70 | 27 |
| Reticulocytes (10^3^/µL) | 1.00 - 34.00 | 11.59 ± 10.03 | 27 |
| Reticulocytes (%) | 0.01 - 0.30 | 0.10 ± 0.09 | 27 |
| WBC (10^3^/µL) | 3.10 - 8.10 | 4.9 ± 1.43 | 27 |
| Neutrophils (10^3^/µL) | 0.70 - 5.60 | 2.50 ± 1.37 | 25 |
| Eosinophils (10^3^/µL) | 0.00 - 0.20 | 0.04 ± 0.06 | 27 |
| Basophils (10^3^/µL) | 0.00 - 0.30 | 0.05 ± 0.08 | 27 |
| Lymphocytes (10^3^/µL) | 1.00 - 4.10 | 2.26 ± 0.65 | 27 |
| N:L ratio | 0.30 - 4.00 | 1.24 ± 0.95 | 25 |
| Monocytes (10^3^/µL) | 0.00 - 0.30 | 0.09 ± 0.07 | 27 |
|  |  |  |  |

**RBC** = red blood cell count; **HGB** = hemoglobin; **HCT** = hematocrit; **MCV** = mean corpuscular hemoglobin; **MCHC** = mean corpuscular hemoglobin concentration; **WBC** = white blood cells.
