# Supplementary material for: Quantifying capture stress in free ranging European roe deer (Capreolus capreolus)
Source: BMC Vet Res. 2017 May 10;13:127. doi: 10.1186/s12917-017-1045-0 (PMC5424289; doi:10.1186/s12917-017-1045-0)
Supplement: Supplementary file 2 — Serum chemistry for free-ranging, non-anesthetized European roe deer (Capreolus capreolus). (DOCX 40 kb) [file 12917_2017_1045_MOESM2_ESM.docx]

**Table S2**Serum chemistry for free-ranging, non-anesthetized European roe deer (*Capreolus capreolus*), captured at Grimsö Wildlife Research Area, included in the study. Ranges as well as the Mean ± SD are presented include all animals.

| Parameter | Range | | Mean ± SD | | *n* | |
| --- | --- | --- | --- | --- | --- | --- |
| ALP (µkat/L) | 0.50 - 1.00 | 0.71 ± 0.15 | | 21 | |  |
| ALT (µkat/L) | 0.50 - 2.30 | 1.04 ± 0.46 | | 21 | |  |
| CK (µkat/L) | 1.30 - 10.60 | 3.55 ± 2.82 | | 21 | |  |
| GGT (µkat/L) | 1.11 - 2.63 | 1.81 ± 0.48 | | 21 | |  |
| GLDH (µkat/L) | 10.00 - 73.00 | 31.1 ± 14.19 | | 21 | |  |
| Amylase (µkat/L) | 0.22 - 0.69 | 0.44 ± 0.10 | | 21 | |  |
| Lipase (µkat/L) | 0.10 - 0.40 | 0.22 ± 0.07 | | 21 | |  |
| Total Protein (g/L) | 57.00 - 90.00 | 68.80 ± 8.63 | | 21 | |  |
| Urea (mmol/L) | 1.70 - 11.50 | 5.65 ± 2.84 | | 21 | |  |
| Creatinine (mmol/L) | 58.00 - 141.00 | 97.66 ± 20.68 | | 21 | |  |
| Cholesterol (mmol/L) | 1.10 - 2.20 | 1.38 ± 0.26 | | 21 | |  |
| Triglycerides (mmol/L) | 0.21 - 0.84 | 0.41 ± 0.13 | | 21 | |  |
| Glucose (mmol/L) | 6.63 - 14.79 | 10.52 ± 2.32 | | 21 | |  |
| Bile acids (mmol/L) | 7.00 - 66.00 | 21.85 ± 14.29 | | 21 | |  |
| Phosphorus (mmol/L) | 1.38 - 3.18 | 2.38 ± 0.45 | | 21 | |  |
| Magnesium (mmol/L) | 0.68 - 1.13 | 0.96 ± 0.11 | | 21 | |  |
| Sodium (mmol/L) | 105.00 - 166.00 | 159.47 ± 4.35 | | 21 | |  |
| Billirubin (μmol/L) | 2.30 - 8.80 | 5.00 ± 1.85 | | 21 | |  |
| Chloride (mmol/L) | 99.00 - 116.00 | 105.57 ± 3.96 | | 21 | |  |
| Iron (mmol/L) | 11.00 - 40.00 | 29.14 ± 9.60 | | 21 | |  |
| Cortisol (mmol/L) | 22.10 - 173.30 | 69.87 ± 33.83 | | 31 | |  |
| Lactate (mmol/L) | 5.10 - 18.90 | 11.7 ± 4.06 | | 22 | |  |

**ALP** = alkaline phosphatase; **ALT** = alanine transaminase; **CK** = creatine kinase; **GGT** = gamma-glutamyl transpeptidase; **GLDH** = glutamate dehydrogenase.
