# Supplementary material for: Quantifying capture stress in free ranging European roe deer (Capreolus capreolus)
Source: BMC Vet Res. 2017 May 10;13:127. doi: 10.1186/s12917-017-1045-0 (PMC5424289; doi:10.1186/s12917-017-1045-0)
Supplement: Supplementary file 3 — Model selection tables. (DOCX 63 kb) [file 12917_2017_1045_MOESM3_ESM.docx]

## Model selection tables

In the model selection tables all models with a ΔAICc < 4 are shown plus the Null-model only containing the intercept. The following variable abbreviations are used for the description of the models: Neu (number of neutrophils), Wait (waiting time; timespan between the arrival of the handling team and the time the animal was taken out of the box), HS (handling score, see methods), RS (release score, see methods), NL (neutrophil/lymphocyte ratio), BT (body temperature), HR (heart rate), Cort (cortisol level), and NULL (the Null-model only containing the intercept).

**Table S3** Model selection table for models testing effects of stress and behavioral score on the LCC-peak (*n* = 24).

| **Model** | ***K*** | **AICc** | **ΔAICc** |
| --- | --- | --- | --- |
| Neu + Wait | 4 | 345.81 | 0.00 |
| Neu | 3 | 348.15 | 2.35 |
| Neu + Wait + RS | 5 | 348.86 | 3.05 |
| Neu + Wait + HS | 5 | 348.99 | 3.18 |
| NULL | 2 | 366.80 | 20.99 |

**Table S4** Model selection table for models testing effects of stress and behavioral score on the LCC–auc (*n* = 24).

| **Model** | ***K*** | **AICc** | **ΔAICc** |
| --- | --- | --- | --- |
| Neu + Wait | 4 | 472.78 | 0.00 |
| Neu + Wait + RS | 5 | 475.46 | 2.69 |
| Neu + Wait + HS | 5 | 475.97 | 3.19 |
| NULL | 5 | 498.77 | 26.00 |

**Table S5** Model selection table for models testing effects of stress and behavioral score on cortisol levels (*n* = 26).

| **Model** | ***K*** | **AICc** | **ΔAICc** |
| --- | --- | --- | --- |
| Wait + RS + Wait:RS | 5 | 243.06 | 0.00 |
| Wait + RS + Wait:RS  + HS | 6 | 244.36 | 1.31 |
| Wait + RS | 4 | 245.81 | 2.76 |
| RS | 3 | 246.20 | 3.15 |
| Wait | 3 | 246.29 | 3.23 |
| Wait + RS + Wait:RS  + HS + Wait:HS | 7 | 246.30 | 3.25 |
| NULL | 2 | 246.88 | 3.82 |

**Table S6** Model selection table for models testing effects of stress and behavioral score on heart rate levels (*n* = 27).

| **Model** | ***K*** | **AICc** | **ΔAICc** |
| --- | --- | --- | --- |
| HS | 3 | 254.95 | 0.00 |
| Wait + HS | 4 | 255.73 | 0.77 |
| Wait | 3 | 256.18 | 1.22 |
| NULL | 2 | 256.38 | 1.42 |
| Wait + HS + Wait:HS | 5 | 256.57 | 1.62 |
| HS + RS | 4 | 257.10 | 2.15 |
| Wait + RS | 4 | 257.25 | 2.30 |
| RS | 3 | 257.66 | 2.70 |
| Wait + HS + RS | 5 | 257.79 | 2.84 |
| Wait + RS + Wait:RS | 5 | 258.01 | 3.06 |

**Table S7**Model selection table for models testing effects of stress and behavioral score on the N:L ratio (*n* = 24).

| **Model** | ***K*** | **AICc** | **ΔAICc** |
| --- | --- | --- | --- |
| NULL | 2 | 67.34 | 0.00 |
| Wait | 3 | 68.44 | 1.10 |
| HS | 3 | 69.58 | 2.24 |
| RS | 3 | 69.72 | 2.38 |
| Wait + HS | 4 | 71.11 | 3.77 |
| Wait + RS | 4 | 71.17 | 3.83 |

**Table S8**Model selection table for models testing effects of stress and behavioral score on the body temperature (*n* = 27).

| **Model** | ***K*** | **AICc** | **ΔAICc** |
| --- | --- | --- | --- |
| NULL | 2 | 61.73 | 0.00 |
| HS | 3 | 63.25 | 1.52 |
| RS | 3 | 63.73 | 2.00 |
| Wait | 3 | 64.27 | 2.53 |
| HS + RS | 4 | 65.72 | 3.99 |

**Table S9**Model selection table for models testing effects of stress and behavioral score on lactate (*n* = 19).

| **Model** | ***K*** | **AICc** | **ΔAICc** |
| --- | --- | --- | --- |
| HS | 3 | 111.20 | 0.00 |
| NULL | 2 | 111.41 | 0.22 |
| Wait + HS | 4 | 113.87 | 2.67 |
| HS + RS | 4 | 114.01 | 2.81 |
| Wait | 3 | 114.10 | 2.90 |
| RS | 3 | 114.14 | 2.95 |
| Wait + HS + Wait:HS | 5 | 114.85 | 3.65 |
